# Socioeconomic Correlates of Healthy Aging Among Older Cambodians

**DOI:** 10.1093/geroni/igaa057.1282

**Published:** 2020-12-16

**Authors:** Kakada Kuy, Yuekang Li

**Affiliations:** Washington University St Louis, University City, Missouri, United States

## Abstract

Healthy aging is one of the most critical goals to attain on the World Health Organization’s global aging agenda for developing countries. However, healthy aging has not been widely studied among the many older adults living in those nations. For example, most of the Cambodia’s economically deprived older adults earn less than a dollar a day, while little scientific evidence is known about their healthy aging situation and their support system. Our study aimed to examine socioeconomic correlates of healthy aging among older Cambodians in three provinces. Data of a sample of older Cambodians ages 60 and above (N=210) from 12 districts were collected. Healthy aging was measured using the Healthy Ageing Index developed based on a Southeast Asian context. We measured social support using the Social Network and Social Support scale. Financial conditions were measured by an index derived from subjective and objective measurements. Multilevel mixed-effects models showed that better social supports from friends and family members, better financial conditions and education were associated with improved health aging among older Cambodians. Supports from friends had a stronger relationship than supports from family members. As one of the first studies examine the social determinants of health among older Cambodians, this study adds to the literature by substantiating the important roles of financial conditions and social support in determining their health and well-being. Findings point to the importance of improving living standard and maintaining social support of the older population in the country.

